# Endoscopic resection of colloid cyst: long-term followup with 63 patients

**DOI:** 10.1186/2045-8118-12-S1-P19

**Published:** 2015-09-18

**Authors:** Albert Isaacs, Walter Hader, Mark Hamilton

**Affiliations:** 1 University of Calgary, Canada

## Introduction

Colloid cysts of the third ventricle are rare, histologically benign lesions that can be associated with obstructive hydrocephalus. Endoscopic removal developed as an alternative to microsurgical craniotomy as a less invasive surgical treatment. This review examines the endoscopic surgical experience for a consecutive series of patients with colloid cyst of the third ventricle.

## Methods

Patients with a diagnosis of “colloid cyst of the third ventricle” who were treated in Calgary between January 1994 and July 2014 were reviewed using a clinic database and registry.

## Results

93 patients were identified. 30 patients without hydrocephalus underwent serial MRI and clinical observation with one patient developing hydrocephalus leading to surgical treatment. 63 patients underwent endoscopic treatment of their colloid cyst (male=34; female=29). The mean age at diagnosis was 46.3 years. 2 patients had been previously treated with other surgical approaches. All surgically treated patients had hydrocephalus and hydrocephalus resolved in all 63 patients. 1 patient sustained an injury to the internal capsule with transient hemiparesis. Mean followup was 8.8 years (range 0.1-20.2 years). 2 patients experienced colloid cyst recurrence treated with a second endoscopic removal.

## Conclusion

Endoscopic treatment of third ventricle colloid cysts can be performed with low risk as an alternative to microsurgical resection.

## References

[B1] Isaacs A M Yuh S J Hurlbert R J Mitha A Penetrating intracranial nail-gun injury to the middle cerebral artery: A successful primary repair Surgical Neurology International 2015 sni_178_1. Submitted 10.4103/2152-7806.166168 PMC4596057 26500798

[B2] Honey C R Yeomans W Jayaraman J Isaacs A Honey C M The Dying Art of Percutaneous Cordotomy in Canada Journal of Palliative Medicine 2014 17 5 PMID: 24717005 24717005 10.1089/jpm.2013.0664

[B3] Paterson R Z Parno T J Isaacs A M Abizaid A Interruption of Ghrelin Signaling in the PVN Increases High-Fat Diet Intake and Body Weight in Stressed & Non-Stressed C57BL6J Male Mice Frontiers in Neuroscience 2013 7 167 PMID: 24062637 24062637 10.3389/fnins.2013.00167 PMC3774989

[B4] King S J Isaacs A M O'Farell E Abizaid A Motivation to Obtain Preferred Foods is Enhanced by Ghrelin in the Ventral Tegmental Area Hormones and Behavior 2011 60 572 580 PMID: 21872601 10.1016/j.yhbeh.2011.08.006 21872601

